# Academic procrastination of medical students: The role of Internet addiction 

**DOI:** 10.30476/JAMP.2020.85000.1159

**Published:** 2020-04

**Authors:** ALI ASGHAR HAYAT, JAVAD KOJURI, MITRA AMINI

**Affiliations:** 1 Clinical Education Research Center, Shiraz University of Medical Sciences, Shiraz, Iran

**Keywords:** Internet, Procrastination, Medical students

## Abstract

**Introduction:**

The internet is an essential and widely used tool for college students; however, high internet dependency can have negative consequences for students, especially regarding academic careers. Such students may tend to postpone their academic tasks. Hence, the current study examines the effect of Internet addiction on academic procrastination among medical students.

**Methods:**

We applied a cross-sectional correlational research design. 233 medical students of Shiraz University of Medical Sciences were selected through convenience sampling and participated in this study. To collect the data, we used two valid and reliable questionnaires. The first was Young's Internet addiction questionnaire (IAT-20), which consists of 20 items based on a 5-point Likert-type scale. The second was Solomon and Rothblum academic procrastination questionnaire, which consists of 18 items based on a 5 point Likert-type scale. We used Pearson correlation, independent T-test, and One-Way ANOVA to analyze the data in SPSS version 22, and considered a significance level of p < 0.05.

**Results:**

Results showed that 57.1% of the respondents were females, and the remaining were males. Findings indicated that 8 (3.43%) of the participants were classiﬁed as severe internet-addicted, and 28.85% of them had a high level of academic procrastination. The results indicated that there was a positive and significant correlation between Internet addiction and academic procrastination (r=0.39, with p<0.01). Also, there was a positive correlation between academic procrastination dimensions (writing a term paper, studying for an exam, keeping up with weekly reading assignments, performing administrative tasks, attending meetings and performing academic tasks in general) and Internet addiction (r=0.22, r=0.32, r=0.21, r=0.29, r=0.33, and r=0.23, respectively, with p<0.01). Finally, the results revealed that male students and those living in the dormitory had a higher level of Internet addiction and procrastination compared to female ones and those living at home (p<0.01).

**Conclusion:**

The findings of the current research reveal that a considerable number of students have levels of Internet addiction and procrastination; the study highlights that students with high levels of Internet addiction are more likely to be at an increased risk of negative outcomes such as insufﬁciently controlled Internet use.

## Introduction

Today, Internet Addiction (IA) is a widespread and universal phenomenon among young people ( 1 ). Also, Internet addiction, recognized as pathological Internet usage (PIU), is defined by extreme or poorly-controlled captivation, desire, or behaviors regarding the Internet use that lead to distress or impairment ( 2 ). Briefly, IA is described by the core signs of Internet overuse ( 3 ). Overutilization of the Internet is related to problems such as depression, anxiety, attention deficit and hyperactivity, and alcohol abuse ( 4 , 5 ), leading to a negative quality of life in terms of health ( 6 ). Studies showed that IA could negatively affect mental health, resulting in loneliness ( 7 ), social isolation ( 2 ), and difficulties in educational-, psychological-, social-, and work-related activities and performance ( 8 , 9 ). 

Research shows that behavioral addictions generally happen during teenage years or young adulthood ( 10 ), so university students are particularly impressionable to IA ( 11 ). They have the global rates of computer and Internet acquisition, and day to day utilization; most of them assign at minimum 2h online every day ( 12 , 13 ). Studies show that Internet addiction has more adverse impacts ( 14 ). For example, in a study, Chou and Hsiao (2000) found that internet addicted students compared to nonaddicted ones revealed more negative outcomes in their academic studies and daily routines ( 15 ). Because of the charm of the internet, most college students report that they spend a lot of time online, while they falter in their task or academic performance ( 16 ). The results of Young’s study (2006) that was conducted on a sample group of teenagers showed the relationship between academic failure and the amount of Internet use. Further, another study found that procrastination in academic activities and schedules because of the excessive use of the Internet can lead to academic failure ( 17 ). 

A widespread phenomenon that interferes with daily and academic tasks is procrastination ( 18 ). As a form of procrastination behaviors, academic procrastination (AP), which is very common among the students ( 19 , 20 ), is an unreasonable trend through which the person delays starting or finishing the academic tasks or other assignments ( 21 ), especially assignments for which deadlines are set. 

The prevalence rates of this problem in students have been reported differently, ranging from 46% ( 20 ), 52% ( 22 ), to 80-95% ( 23 ). Therefore, procrastination should not easily be overlooked, but it should be taken seriously because of its increasing prevalence between the students and even in society ( 24 ). Numerous studies have shown that procrastination behaviors are related with lower academic achievement (e.g. poor grades) ( 20 , 22 ) and higher course withdrawal ( 20 ), anxiety, and decrease of self-confidence ( 25 ). Briefly, procrastination is detrimental to both academic performance and mental health ( 25 ). Further, Gustavson and Miyake (2017) reported that students with high levels of procrastination were unable to organize and achieve their academic goals ( 26 ). 

Although the negative consequences of procrastination in an individual's daily life might not be considered, the outcomes of its prevalence between medical students who are to take significant responsibilities in the future can be irreparable. Therefore, it is important to recognize the prevalence of AP and its essential antecedents among medical students. In this regard, some studies have confirmed the robust relationship between internet usage and procrastination. For example, Davis et al. (2002) reported a strong, positive relationship between procrastination and PIU. Further, Kandemir (2014) showed a strong, positive correlation between AP and the degree of IA ( 17 ). Even though some studies have been conducted in American and European contexts on IA and its relation with AP, Kljajic and Gaudreau (2018) believed that surprisingly few studies had been carried out in Asian context ( 27 ), specifically in Iran and on medical students.

On the other hand, the results of a meta-analysis conducted recently showed that the frequency of IA amongst medical students was nearly five times that of the general population ( 28 ). Based on such results, the researchers believe that medical students are vulnerable to internet addiction, and efforts should be taken to increase awareness and prevent this problem and its consequences in them ( 29 ). Thus, the present study aims to examine the relationship between IA in medical students and their AP.

## Methods

### *Study design and participants*

The present research is a quantitative cross-sectional research design in which 340 medical students from all the periods of the medical program at Shiraz University of Medical Sciences were enrolled to complete an anonymous self-report questionnaire. The sample size was determined according to Krejcie and Morgan's sample size determination table and through convenience sampling method. The response rate was 243/340 (71%). After cleaning and removing the invalid and missing data (no response to many items or the same response to each item), 233 questionnaires remained. The inclusion criteria included medical students at Shiraz University of Medical Sciences and willingness to participate in the study. Also, exclusion criteria included incomplete response to the questionnaires and unwillingness to participate in the study. 

### *Measures*

To collect the data, we used two valid and reliable questionnaires.

### *Young's Internet addiction questionnaire*

One of the most frequently used questionnaires to assess excessive or pathological use of the internet is Young's Internet addiction test (IAT), which consists of 20 items. This scale was designed on a 5-point Likert-type scale ranging from 1 (rarely) to 5 (always). IAT mentions several issues related to excessive Internet utilization, such as loss of control of using the Internet, leading to neglecting work and relationships, addictive symptoms (i.e. craving) when being online, etc. ( 30 ). The higher score on the questionnaire indicates greater addiction on the internet and the severity of the problems that individuals experience as a result of overuse. The total score of the IAT ranges from 20 to 100 and represents an individual's tendency to or the degree of IA ( 31 , 32 ). An overall score of less than 30 indicates a normal level of Internet usage, while a score between 31 and 49 represents mild level addiction, between 50 and 79 indicates moderate addiction, and that higher than 80 indicates a severe level of Internet addiction ( 16 , 33 ). Most studies in different countries have used this scale to assess IA ( 3 , 31 , 34 , 35 ). The test-retest reliability (r = 0.85) and internal consistency (α = 0.90–0.93) of the IAT was approved in primary examination ( 31 ). Therefore, the IAT has good psychometric properties and represents the key diagnostic criteria of IA ( 32 ). The validity and reliability of IAT have been reported at an acceptable level in Iran ( 36 , 37 ). In the present study, the questionnaire’s face and content validitites were approved by a panel of experts. Also, its reliability was measured using Cronbach’s alpha and composite reliability, which were 0.89 and 0.90, respectively. 

### *Procrastination Assessment Scale – Students*

Another scale we used was Procrastination Assessment Scale – Students (PASS). This scale was made by Solomon &amp; Rothblum (1984) and contained two parts; in the current study, we used only the first part which assesses the medical students’ procrastination in six domains of 1) studying for an exam, 2) writing a term paper, 3) performing administrative tasks, 4) keeping up with weekly reading assignments, 5) attending meetings, and 6) performing academic tasks in general. Each dimension was assessed through three questions and overall 18 questions with a 5-point Likert scale. In the current study, we used the first two items in each domain. The first item assesses the frequency of procrastination, and the second one measures how much it causes problems in one’s performance of duties. Therefore, the amount of AP, as well as the problems it causes for the students ranges from 2-10 in each dimension. Also, for all dimensions, the total score ranged from 12 to 60. The higher the score, the higher the AP. The internal consistency of PASS was reported at a good level by Solomon and Rothblum (0.84) ( 20 ). Its validity and reliability were approved in many studies in Iran ( 38 , 39 ). In addition, the face and content validities of the PASS were approved by a panel of experts. The reliability was measured using composite reliability and Cronbach’s alpha, which were 0.88 and 0.86, respectively. 

### *Ethical Considerations*

First, we obtained ethical approval through the Ethics Committee of Shiraz University of Medical Sciences; then, we obtained the participants’ written informed consent and asked the students to complete the anonymous questionnaires. 

### *Data analysis*

To analyze the data, we used descriptive and inferential statistics (Pearson correlation coefficient, Independent T-test, One-Way ANOVA). Also, we used SPSS version 22 (IBM Corp, Chicago) to analyze the data. 

## Results

First, to test the normality, we used the Kolmogorov-Smirnov (K-S) test, and skewness and kurtosis test; the result showed our data was normal. Then, we used the parametric tests. In terms of demographic findings, 57.1% of the respondents were females, and 42.9% were males. Of the 233 students, 8 (3.43%) participants with IAT score ≥ 80 were classiﬁed as severe internet addicted. In the case of procrastinator behaviors, the finding revealed that 28.85% of the students always or nearly always indicated a high level of procrastination. Based on the results, 33.5% of medical students reported that their procrastination in academic duties had been problematic for them. 

The Pearson correlation results showed that IA had a positive and significant correlation with AP and its dimensions (p≤ 0.001) (Table 1). 

**Table 1:** The correlation between the students’ IA and AP

	Writing a term paper	Studying for an exam	Keeping up with weekly reading assignments	Performing administrative tasks	Attending meetings	Performing academic tasks in general	AP
IA	0.22**	0.32**	0.21**	0.29**	0.33**	0.23**	0.39**
Mean± SD	3.09±0.88	3.11±0.99	2.86±0.84	3.21±1.0	2.93±0.87	2.76±0.86	2.99±0.63
P Value	0.001	0.001	0.001	0.001	0.001	0.001	0.001

** p < 0.01

Based on the results, there was a significant difference between the male and female students regarding the level of AP, so that the female ones were procrastinator less than the male students. Also, the finding showed that the students who lived in a dormitory had more procrastinator behaviors than those who did not (Table 2). 

Another result indicated that there was a significant difference between the female and male students regarding IA, so that the female students were at a lower level of IA than the male ones. Other findings indicated that the students who lived in a dormitory had more levels of IA than those who did not (Table 2).

**Table 2:** Comparison of AP and IA in the students

	Gender	Mean± SD	t-test	P
AP	Male	3.10±0.62	3.03	0.01
	Female	2.85±0.62		
	Dormitory residing students	3.23±0.58	6.04	0.01
	Home residing students	2.76±0.52		
IA	Male	3.03±0.56	6.45	0.01
	Female	2.57±0.52		
	Dormitory residing students	2.95±0.58	3.06	0.01
	Home residing students	2.72±0.59		

The result of One-Way ANOVA revealed there was a significant difference between educational levels regarding IA (P≤ 0.05). Thus, we used the Tukey HSD test to compare the groups, and the results showed that the mean score of IA for the students who were at the studentship period(M=2.95, SD=0.57) was significantly higher than those who were at basic sciences period (M=2.67, SD=0.58). Also, other results from One-Way ANOVA showed that there were no significant differences in AP among students at different levels of education (P≥ 0.05) (Table 3). 

**Table 3:** Summary of the results of One-Way ANOVA

	Educational Level			
		Mean±SD	F	P
AP	Bsc	2.83± 0.61	2.11	0.080
	Phy	3.01± 0.85		
	Stu	3.11± 0.50		
	Ext	3.03± 0.64		
	Int	3.05± 0.70		
IA	Bsc*	2.67± 0.58	2.42	0.049
	Phy	2.81± 0.59		
	Stu*	2.95± 0.57		
	Ext	2.91± 0.60		
	Int	2.92± 0.53		

Bsc (Basic Sciences), Phy (Physiopathology), Stu (Studentship), Ext (Externship), Int (Internship).

* Groups with significant differences

## Discussion

In this study, the relationships between IA and AP were examined. According to the hypothesis, a positive correlation exists between IA and AP among medical students. This means that students with a higher level of IA are more liable to AP. The result is consistent with the studies performed in another context ( 14 , 17 , 34 , 40 , 41 ). According to Young (1998), when the Internet dominates people's lives, it can be explained by the concept of preoccupation. The preoccupation caused the individual to put his/her responsibilities and duties such as career, education, occupations, and house into the second priority. The Internet becomes the heart of an individual’s life, and in turn, it causes students to delay their academic tasks ( 30 ).

Besides, Blunt and Pychyl (2005) believe that when the assignment is considered as intrinsically unpleasant or less enjoyable, a person is more likely to procrastinate ( 42 ). For example, a task that is perceived as boring, difficult, and unpleasant causes a person to avoid from doing it. Conversely, the Internet with providing many entertaining interferences is characterized as a tool by which a person could obtain an interesting, pleasant, and entertaining experience beneficial to perceived stress relief ( 43 ). Inherently, the Internet is considered as a distractor and an activator for procrastination ( 40 ). This is the case, especially for Internet addicts who can not resist the attractions of online entertainment and, thus, devote more time to participation in online activities, resulting in additional procrastination ( 14 ).

The results of the study showed that male students had a higher level of IA than females in general. Many studies suggest that males have higher prevalence estimates of IA ( 44 ), Overall, results from different areas of addiction also indicate that women are less susceptible to addictive behaviors ( 45 ). This tendency also exists in many behavioral addictions, with many studies suggesting, for example, a higher prevalence of IA among males than females ( 46 ). A new review of seven studies across different cultural groups showed that most findings revealed males at higher apparent risk for IA, with the difference in gender-related prevalence estimates of IA increasing over time ( 47 ). This pattern can reflect male trends to use applications with potentially high IA risk (e.g. online games or cyber sexual activities) ( 48 ). Moreover, males score higher than females on potential risk factors (e.g. maladaptive cognitions) and lower on IA-related protective factors (e.g. effortful control) ( 49 ). Also, females (especially adolescents) regularly obtain more family control than males, which can help them to avoid spending too much time on the internet ( 50 ). 

One of the important determinants of addictive behaviors is availability. In some cultures, male superiority in IA prevalence may be related to high internet availability in males than females. According to the International Telecommunication Union (2016), the men's’ internet use rates are higher than women in nearly all regions of the world. The gap between internet user gender grew from 11% in 2013 to 12% in 2016 (cited in 50). Also, another explanation might be sociocultural customs that is a factor that limits women from accessing and using the internet.

According to the results, there was a significant difference between the students who live in the dormitory and those who do not in the level of IA; in other words, those residing in the dormitory were more addicted than those who did not. This may be because of free, easy, and cheap access to the Internet for the students who live in the dormitory rather than those who do not. Also, students who do not live in dorms, especially female ones, are freer and do not have parental supervision, which may increase their willingness to overuse the Internet.

It was also found out that male students had a higher level of procrastination than females. This finding is in line with the results of previous research ( 22 , 51 ). Some researchers believe that girls are significantly higher in learning focus, planning, study management, motivation, and persistence than boys ( 52 ), which results in less procrastinating behavior. Generally, in academic contexts, female students perform more competitively than male ones and are more motivated to gain higher scores, so they have less AP. Some researchers believe that females are more scary for obtaining lower scores than males, so they make an effort to keep away from it ( 22 ). Therefore, it can be said that fear of failure acts as a mechanism for the lower level of procrastination in females.

According to the results, there was a significant difference between the students who live in the dormitory and those who do not in the level of AP; in other words, those residing in the dormitory were more procrastinator than those who did not. The atmosphere of the dormitory causes the students to spend more time on pastime and hobbies together; also, they spend much of their time on the Internet and social networks (as the results of our study showed); this leads to postponing their academic tasks. It is obvious that the dormitory context, unlimited access to the Internet, and their hobbies lead to negative outcomes as to their education and performance.

Researches have shown that interventions have been effective in reducing Internet addiction, so the following suggestions can be applied to reduce Internet addiction and have a positive impact on reducing student procrastination.

- Identifying students who are potentially at risk of Internet addiction and trying to effectively intervene, because some studies have shown that some risk factors (anxiety disorders, social phobia, hyperactivity, impulsivity, and introversion) affect Internet addiction.
- Trying to develop specific skills for preventing Internet addiction, including the decrease of the positive consequence belief of Internet usage, self-efficacy, self-control, or self-denial from addictive online applications.
- Develop skills associated with students' daily regime and use of free time, such as keeping a sleep schedule, carrying outgroup activities and free-time activities, and encouraging students in creative, exploratory, and exciting healthy activities.
- Holding workshops on Internet addiction for students and ways to control this behavior.

## Conclusion

This study had some limitations. First, because the present study used correlational design, it did not prove the causal relationship between IA and procrastination. The results do not show that IA causes procrastination or vice versa. To overcome this, more lab experiments and longitudinal studies are suggested for future studies. Secondly, the sample of the present study was restricted to medical students, so generalizing the results is limited. Also, the results of this study are based on the qualities of self-report measures. Other studies may explore the causes and consequences of IA and procrastination for students with systematic analysis through in-depth observations and interviews. 
